# Supplementary material for: Association between oral microbiome and seven types of cancers in East Asian population: a two-sample Mendelian randomization analysis
Source: Front Mol Biosci. 2023 Nov 21;10:1327893. doi: 10.3389/fmolb.2023.1327893 (PMC10702768; doi:10.3389/fmolb.2023.1327893)
Supplement: Supplementary file 1 [file DataSheet1.ZIP › Supplementary Materials/MR plots for tongue/tongue═╝/Pancreatic cancer/╥╚╧┘░⌐_╔╕╤í╜ß╣√/pheno.1357_to_pancreatic cancer_leave_one_out.pdf]

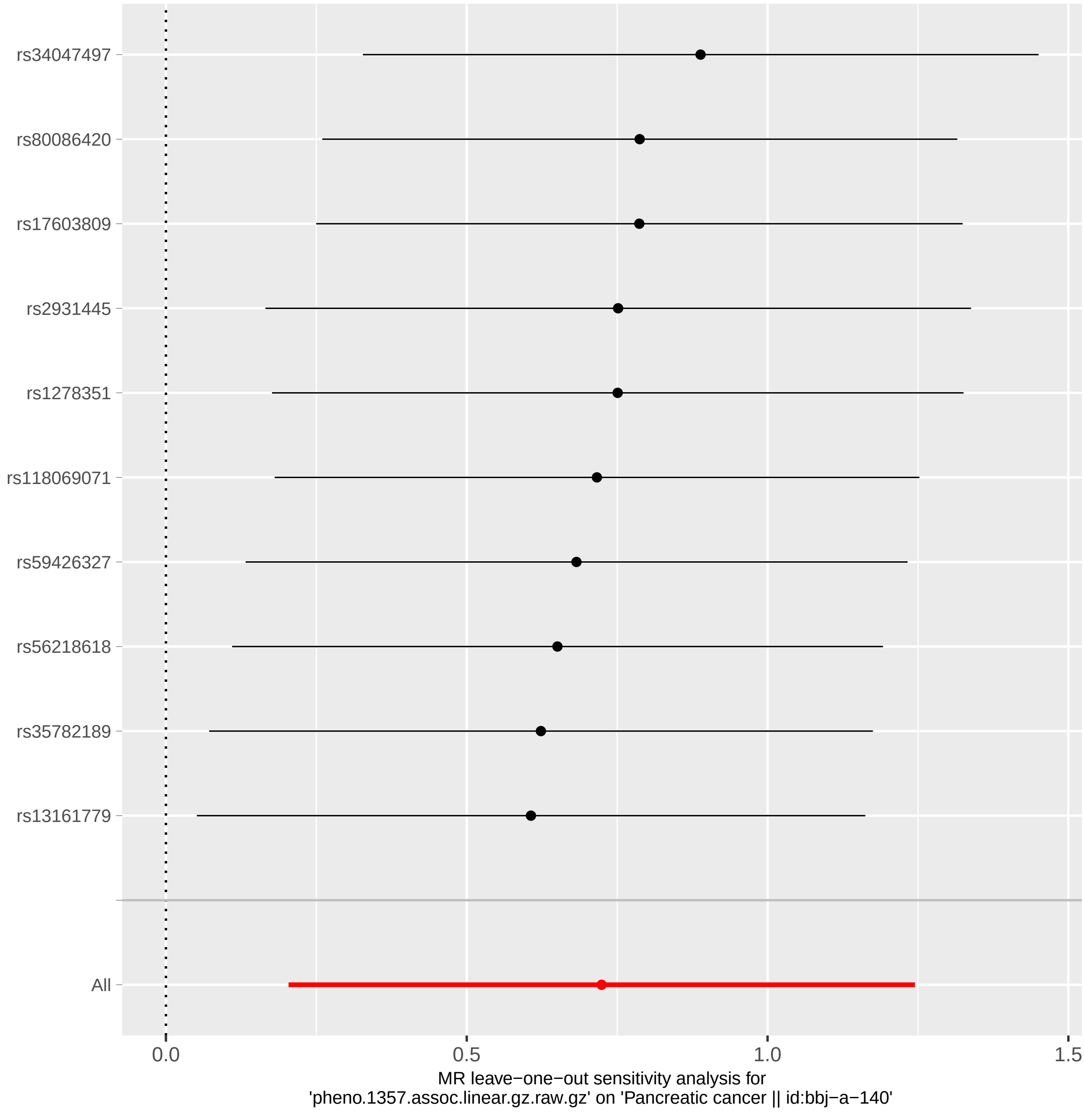

rs34047497
rs80086420
rs17603809
rs2931445
rs1278351
rs118069071
rs59426327
rs56218618
rs35782189
rs13161779
All
0.0
0.5
1.0
1.5
MR leave−one−out sensitivity analysis for
'pheno.1357.assoc.linear.gz.raw.gz' on 'Pancreatic cancer || id:bbj−a−140'
